# The Needs and Backbone of the Hospital System

**Published:** 1888-05-19

**Authors:** Randolph Churchill


### The Needs and Backbone of the Hospital System.

By the Right Honourable Lord Randolph Churchill, M.P.

(Being his speech at the festival dinner of St. Mary's Hospital, held on Saturday, May 12th.)

I could wish—and I am resorting to no mere formality of expression when I say that I greatly desire—that the toast of the evening had been entrusted to abler and more experienced hands than mine, because I am sensibly aware that the life of a politician tends more and more day by day to run into a regular and undeviating groove, and that the incidents in the avocation of a political life have a marked tendency to narrow both the form of action and breadth and depth of thought which are necessary to the performance of duties such as mine of this evening. But I claim your indulgence, I claim the indulgence which I think you ought to extend to an ordinary member of Parliament dealing with matters somewhat out of his line; and at the same time I frankly warn you that with your permission I shall make even a larger demand upon your patience than I intend to make on your pockets. We are gathered together this evening in order, if possible, to promote and to increase the efficiency of our London hospital system. Now, it is perhaps advantageous to reflect upon the nature of our London hospital system. The best way of describing it is perhaps to say that it is utterly unlike any system which obtains in the foreign countries of the European continent. The system of hospital administration in foreign countries is mainly, I think, State-aided; either State-aided or municipally-aided, and either under State control or under municipal control. I do not think that there is anyone in this country who wishes to copy the foreign system of hospital administration in that respect. If we want to see the evils of the rate-aided system exemplified in a sufficiently glaring manner we have only to study the proceedings and the policy of the Metropolitan Asylums Board, and such a study would not encourage us to depart from the system which obtains in this country.

What is the system which obtains in this country? What are its main features? Its main features are that it is voluntary, it is free, and it is independent; and it possesses all that vigour and all that enterprise which we in this country rightly imagine attaches to free and independent institutions. Having said that, I think you will agree that it follows that we should be wrong if we did not recognise practically that our free and voluntary system, with all its merits, is attended with some disadvantages. Those disadvantages may, perhaps, best be realised by reflecting for a moment on the extent of the London hospital system. That extent is well shown in two ways. In the first place it is well and forcibly shown by the expenditure which takes place under it. I believe I am right in saying that that expenditure amounts, according to the latest returns, to a sum of about £650,000 a-year. That is a large sum, gentlemen, for the hospitals of one city, and, in connexion with the expenditure, we may also usefully reflect on the income. I believe I am right in saying that the income the hospitals in London enjoy is something like £560,000 a-year, which shows undoubtedly a deficiency of income as compared with expenditure—a deficiency which is to some extent made up by legacies, which vary from £50,000 to £100,000 a-year.

Now, although legacies as a form of hospital income are by no means to be under-rated, still we must recollect that they are capricious in their incidence, and fluctuating in their amount, and it would be practical to estimate what I believe is a fact, that the general deficiency of income over expenditure which the London hospitals have to meet may be stated at a figure of something like £100,000 a-year. That shows the extent of the London hospital system. Its extent is also graphically displayed by the population with which the London hospital system attempts to deal—by the population and area with which we are interested. The population of the metropolis numbers, I believe, some 4,215,000 people, and out of this 4,215,000, if we may except all those people who receive assistance from the Metropolitan Asylums Board, and all those who receive assistance from the poor-law infirmaries, we find that 1,300,000 receive direct relief from our London hospital system in the metropolis—a proportion of about one in four, and we must remember that that figure does not include the very large number of persons who come up from the country to receive the skilled medical treatment of the metropolis.

Now, the system of hospital administration in London is, as I have said, a voluntary one, and it is confronted by a difficulty—a stupendous difficulty—which is embarrassing, and will probably baffle all the devices of statesmen, and will confound all the conjectures of philosophers. I allude to the great and rapidly-increasing size of the metropolis. The metropolis is outgrowing not only all its capacity for local government — not only, perhaps, even its capacity for ordinary locomotion—but undoubtedly is out-growing its hospital system. That, gentlemen, is well shown by the situation of the bulk of the London hospitals, and it is a most remarkable fact that the large majority of those hospitals are situated within a radius of about one and a-half miles from Charing Cross; for out of 6,500 occupied beds which the London hospitals provide, 4,500 are within a radius of a mile and a-half of that centre. Now, what is the radius of the area which the hospital system of London has to deal with? Its area extends no less than seven miles from Charing Cross. You will see that that fact at once suggests the reflection of the startling disadvantage which arises from the present localisation of our hospitals in London. It is an evil, a disadvantage which I think cannot possibly be neglected, and it will be absolutely necessary before long that great efforts should be made to move some of our large hospitals out of their present narrow areas into areas where they can work with more effect. This is the fact, and it must be borne in mind that only 1,560 occupied beds in hospitals remain for the use of the population inhabiting the outer ring, which outnumbers the population of the inner ring in the proportion of something like three to one. These may seem dry statistics, but they have a marvellous practical effect upon the daily life of London, if you think for a moment what they really mean—what it really means to fail, on the part of the working-classes, to obtain rapid and easy access to the hospitals. It very often means—in numberless cases it means—the speedy and possibly fatal progress of disease; and very often means—and probably in numberless cases means—great misery, and utter ruin accruing to those who are dependent upon the bread-winning exertions of the sick.

I own, my lords and gentlemen, that I am terribly shocked and startled at the condition of our modern society, when I reflect upon, and when I contrast, what I have called in another place the fatal facility of recourse to the public-house, and the desperate difficulty of recourse to hospital treatment; and when I remember the eager and wild enthusiasm which is evoked in the defence of the one, and the apathy which almost paralyses for a time when you endeavour to mitigate the other. There is another disadvantage attaching to our voluntary system which we shall do well to bear in mind. It is not so much, I am informed on good authority, that the hospital accommodation in itself is inadequate, though doubtless much might be done profitably to extend it; but it is that the funds at the disposal of the hospital authorities are decidedly inadequate to the duties which are cast upon them. Just imagine the remarkable state of things shown by the fact that out of 9,372 hospital beds only 6,702 were occupied last year. It is not at all for want of cases to occupy these beds, but the fact is that the funds at the disposal of the hospital authorities do not enable these beds to be occupied.

I have spoken of the great deficiency in London hospital funds, amounting to £100,000 a-year. What is the feature of that deficiency? The main feature, and the deplorable feature, of that deficiency is the smallness of the annual subscriptions to the hospitals. I suppose I shall carry you with me when I say that the annual subscriptions are the backbone, the fundamental basis of the resources of London hospitals. Now, how does the matter stand? I regret to say, but I think it my duty to say, that there is absolutely a falling-off, a relative falling-off in the annual subscriptions to hospitals in the metropolis. If I take the expenditure of the year 1877 and the income of that year, I find that the income from subscriptions derived by fourteen great London hospitals amounted to £32,544, and their expenditure amounted to £201,683. If I take the year 1887, I find the income has increased to £35,667—a poor and paltry increase of something like £3,000; but I find that the expenditure has increased to a sum amounting to £247,800, a large increase of nearly £50,000 a year in expenditure; so that whereas the percentage of income derived from subscriptions upon expenditure was in 1877 something like 16·1, it has positively fallen off in ten years to a percentage amounting to 14·4 in 1887. That is not, I venture with great confidence to say, a satisfactory state of things, and it shows there is a serious falling off in the efforts of the rich in support of these great institutions.

I understand that there are many who would say that this hospital question is more a question which concerns the masses of the people, and that those people should do a good deal to help themselves. Well, I think that is a perfectly fair observation. The question of hospital administration is essentially a people's question, and the sufficiency or insufficiency of hospital accommodation enters into the daily life of the masses of the people in an extremely acute form; and it is not unprofitable to inquire what the masses of the people are doing for themselves in this matter. They are making, I am glad to say, most marked efforts, which I am pleased to be able to bring to your notice. If I look at the Hospital Saturday Fund, which is recognised to be a popularly started and a more popularly contributed fund than the Hospital Sunday Fund, I find that the Hospital Saturday Fund already amounts in the metropolis to 25 per cent. of the Hospital Sunday Fund, but this is not all. I spoke of the inconvenient localization of the London hospitals for the working-classes of the metropolis. I can now show that the working-classes have endeavoured to remedy this, for in the north of London the Great Northern Central Hospital has been founded to a very large extent by the contributions of the working-classes. From the district of Islington alone no less a sum than £5,000 was collected from the mass of inhabitants of that district; and in the crowded districts of East and West Ham a hospital has been started by similar efforts, and is being attended with similar results. But, gentlemen, if I turn your attention from the metropolis to the provinces you will find that the efforts of the working-classes there are most marked. The Hospital Saturday Fund in Birmingham,—a town, I believe, which can proudly claim the honour of having originated both these Funds,—already exceeds and largely exceeds in amount the Hospital Sunday Fund, and whereas the Sunday Fund is decreasing there, the Saturday Fund is increasing. But more than that, if I turn to a great town like Glasgow, I find the artizan classes of that great city contributing no less a sum than £5,000 a-year to the support of the Royal Infirmary of Glasgow, and in good and prosperous years they have been known to contribute as much as £11,000. Another district of England which I may refer to in support of my argument is that of the Potteries, and I am informed that at Stoke, with respect to the North Staffordshire Infirmary, the working class organization there has such an admirable system among themselves that they practically pay for all the treatment received in that hospital by any of their body, and also give £500 a-year as a free gift to the institution.

These, you will agree with me, are really noble efforts, which deserve to be signalized, commended, and encouraged, and brought to the notice of the public. But I would like to stimulate your metropolitan activity by contrasting the efforts of the provinces with regard to subscriptions to hospitals with the efforts of this great town, and I may state that if you take the London Hospital, a hospital working in one of the poorest districts of London—Whitechapel—the subscription revenue of that hospital only amounts to five per cent. of its expenditure. Turn to Leeds; the subscription list of the great hospital there amounts to no less than 45 per cent. of its expenses. I think you will admit that I have placed before you good arguments in support of my contention that there is a deplorable and serious falling off in the subscriptions to metropolitan hospitals, and when you recollect the terrible anxiety and labour which are thrown on the managers of a hospital by the serious fact of having to face a bad subscription list, and how much more profitably the time of those gentlemen who manage our hospitals might be occupied than in continually begging and the worry of having to devise means to cover the deficiencies which the subscription list shows, I think you will agree with me when I say that one great public effort should be made to stimulate and render more assured and certain the income of our hospitals.

I cannot think, nor do I think, anyone will be of opinion that the rich of the metropolis are doing anything like what they ought to do. I have no doubt there are many rich people thoughtless, and some, perhaps, extremely selfish, who say to themselves, "Why should I give any money to hospitals? The hospitals are nothing to me; I am never likely to go to a hospital." Now, can you conceive a reflection which displays a more dreadfully thoughtless state of mind? I suppose that to you I should be stating a truism, but it is a truism which is either greatly forgotten or not known to many outside this room, that if it were not for the hospitals we should positively have no doctors at all. The hospitals of this metropolis and of the great towns of England are a perpetually-flowing fountain of medical science, and all great discoveries of medical science, all the new remedies which medical science brings to light for the treatment of disease, and all the ingenious associations of the appliances of mechanism and of instruments, take their origin and thrive in the hospitals before they are applied to the treatment of disease outside the hospitals. And not only if we had no hospitals—if there was such a convulsion of society that there were no hospitals—not only should we have no doctors, but we should not have what is no less necessary—we should have no supply of trained nurses; and all medical men who may be present will agree with me that in many forms of disease the trained nurse is as essential to recovery, and in some forms of disease more essential to recovery, even than the doctor himself. This is the fact, that for the treatment of all these numerous forms of disease, of illnesses which seem to be really increasing in our modern society, many of which the rich bring upon themselves by their over-indulgence in luxuries or over-refinement of sensuality, the rich and wealthy classes owe everything they receive to the hospital system. I wonder, gentlemen, how many rich people there are in this town—and I hope some of them will have the opportunity of reading what I say—I wonder how many people there are in this town who have met with serious illness or accident, and who, by almost miraculous medical skill and almost miraculous patience in medical nursing, have recovered, who never thought to give a single sixpence to the hospital to which they may be said to owe absolutely their prolonged life.

To bring all these general remarks on our London hospitals to a conclusion, I would say that the needs of the London hospital system appear to me to be twofold. They are, in the first place, the want of funds—the want of enlarged funds—the want of largely-enlarged funds ; and, secondly, the want of improved and enlarged hospital sites. I think I may say, with regard to the needs for enlarged funds, that at the very least the sum of £200,000 a-year ought to be given to London hospitals in addition to what is now given. I believe at least £100,000 a-year is required for the efficient performance of the work which the London hospitals are bound to take in hand, and that at least another £100,000 is required for a work which ought to be done, which requires to be done, but which for want of resources is not done.

I turn to the other need which persons of authority tell me of—the need for large and improved sites of London hospitals. There are, I believe, many hospitals in London which are greatly, and I believe injuriously, affected by the cramped positions which they occupy. They are crowded up in narrow places, they are shut off from the constant flow of fresh air ; they are surrounded in many cases by narrow and dirty streets and lanes ; and they are to a large extent hidden away from public notice. Now this, I think, is a most crying shame. All will agree that a great hospital ought to occupy, in a great town like this, not only upon hygienic grounds, but on grounds of public convenience and of public honour, a prominent position in our principal thoroughfares. Our hospitals ought to be able, by their own position and by their general outside appearance, to bring themselves forcibly every day to the constant notice and attention of the public. That, I regret to say, is by no means the case, and here I am glad to signalise what I may call a millionaire's opportunity. Let all millionaires, and I don't know whether there are any in this room, get an ordnance map and let them study the sites of the London hospitals, and let them see where they can buy land to enlarge those sites and give better air, and let them buy that land and give it to the hospitals in question. I have a millionaire in my eye—I won't mention his name, but I am sure he will recognise himself—he is a millionaire who possesses a surprising number of millions, and I can most truthfully say that if I were in his place I would not rest quiet until I had devoted at least one million to the improvement of the sites of the London hospitals.

